# Supplementary material for: Identification of photoperiod-induced specific miRNAs in the adrenal glands of Sunite sheep (Ovis aries)
Source: Front Vet Sci. 2022 Jul 22;9:888207. doi: 10.3389/fvets.2022.888207 (PMC9354845; doi:10.3389/fvets.2022.888207)
Supplement: Supplementary file 1 [file Data_Sheet_1.ZIP › Supplementary file/miRDeep2 file.zip!/miRDeep2 file/Know_Structure/SPLP42A3/oar-mir-1197.pdf]

miRBase precursor : oar-mir-1197
Total read count : 56
oar-miR-1197-5p read count : 4
oar-miR-1197-3p read count : 28
remaining reads : 24

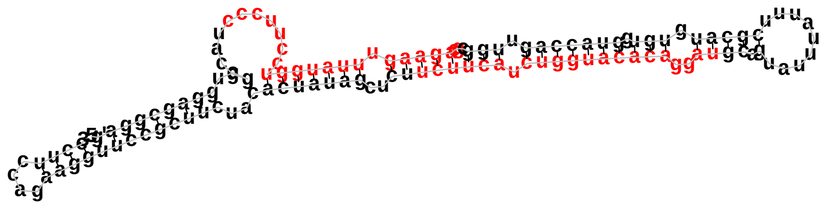

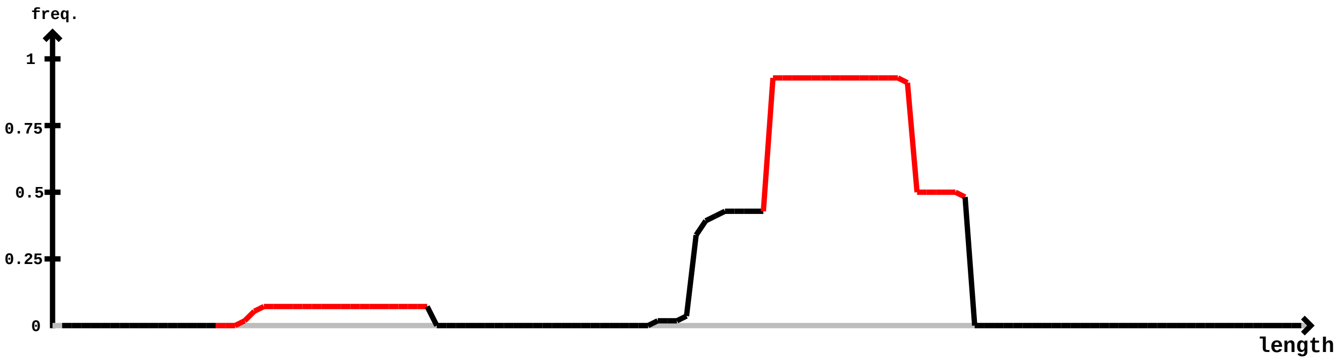


```
                                                                                 oar-miR-1197-3p
                 oar-miR-1197-5p
5'- gaggcgaggucggcaucccuuccugguauuugaagacgcgguugaccauggugugua cgcuuuauuuaugacguaggacacauggucuacuucuucucgauaucacaucuucgccuuggaagaccuucc  -3'   exp
```

```
5'- gaggcgaggucggcaucccuuccugguauuugaagacgcgguugaccauggugugua cgcuuuauuuaugacguaggacacauggucuacuucuucucgauaucacaucuucgccuuggaagaccuucc  -3'   exp
```
